# Walkability im Alter – Umweltfaktoren der Wohnumgebung und funktionssensitive Messung im urbanen Raum

**DOI:** 10.1007/s00103-026-04288-6

**Published:** 2026-08-20

**Authors:** Daniela Koller, Eva Grill, Ralf Strobl

**Affiliations:** 1 Institut für medizinische Informationsverarbeitung, Biometrie und Epidemiologie, Medizinische Fakultät, LMU Medizin, Ludwig-Maximilians-Universität München, München, Deutschland; 2 Muskuloskelettale Versorgungsforschung, Klinik für Orthopädie und Unfallchirurgie, LMU Klinikum, LMU Medizin, Ludwig-Maximilians-Universität München, Marchioninistr. 15, 81377 München, Deutschland; 3 Deutsches Zentrum für Schwindel und Gleichgewichtsstörungen, LMU Klinikum, LMU Medizin, Ludwig-Maximilians-Universität München, München, Deutschland

**Keywords:** Gesundes Altern, Körperliche Aktivität, Funktionsfähigkeit, Mobilität, Umweltgerontologie, Healthy aging, Physical activity, Functioning, Mobility, Environmental gerontology

## Abstract

Walkability beschreibt die Gehfreundlichkeit einer Nachbarschaft und ist ein zentrales Konzept zur Förderung von Mobilität, körperlicher Aktivität und sozialer Teilhabe im Alter. Bestehende Ansätze bilden die spezifischen Bedürfnisse älterer Menschen bislang nur unzureichend ab, insbesondere bei funktionellen Einschränkungen. Der Beitrag zeigt, wie Walkability im höheren Lebensalter konzeptionell weiterentwickelt, funktionssensitiv erfasst und in einem urbanen Kontext empirisch untersucht werden kann.

Vorgestellt wird ein Operationalisierungsframework für Walkability im höheren Lebensalter, das auf Vorarbeiten der Autor:innen und einer Delphi-Konsensstudie basiert. Es umfasst die Dimensionen Wohnumfeld und Verkehrsinfrastruktur, Erreichbarkeit von Zielen und Begegnungsorten sowie Attraktivität und Sicherheit der Umgebung. Zentrales Merkmal ist die systematische Berücksichtigung der Interaktion zwischen funktionellem Status und Umweltanforderungen.

Am Beispiel des geplanten WALK-Projekts in München wird die praktische Anwendung der Indikatoren in einer kommunalen Mixed-Methods-Studie beschrieben. Kombiniert werden qualitative Interviews, Walk-&-Talk-Begehungen, Expert:innenbefragungen und GIS(geografische Informationssystem)-basierte Analysen. Ziel ist es, Barrieren und Förderfaktoren des alltäglichen Gehens älterer Menschen zu identifizieren und daraus Empfehlungen für eine gesundheitsfördernde, alterssensible Stadtentwicklung abzuleiten.

## Abstract (en)

Walkability describes how supportive a neighbourhood is for walking and is a key concept for promoting mobility, physical activity and social participation in older age. However, existing approaches do not adequately reflect the specific needs of older adults, especially in the presence of functional limitations. This article outlines how walkability can be further conceptualised, measured in a function-sensitive way and empirically examined in an urban context.

The paper presents an age-specific operationalization framework for walkability based on the authors’ previous work and a Delphi consensus study. It includes three dimensions: residential environment and transport infrastructure, accessibility of destinations and meeting places, and attractiveness and safety of the environment. A central feature is the systematic consideration of the interaction between functional status and environmental demands.

Using the planned WALK project in Munich as an example, the article illustrates how these indicators can be applied in a municipal mixed-methods study. The design combines qualitative interviews, walk-and-talk assessments, expert interviews and GIS-based analyses. The aim is to identify barriers and facilitators of everyday walking among older adults and to derive recommendations for health-promoting, age-sensitive urban planning.

## Hintergrund

### Gesundes Altern und die Bedeutung der Umwelt

Durch den demografischen Wandel, gekennzeichnet durch steigende Lebenserwartung einerseits und das Älterwerden der geburtenstarken Kohorten andererseits, wird der Anteil älterer und hochaltriger Menschen in der Bevölkerung in den nächsten Jahrzehnten deutlich ansteigen [1, 2]. Mit zunehmendem Alter steigt auch das Risiko für chronische Erkrankungen, wie kardiovaskuläre Erkrankungen, Krebs, Demenzen oder Arthrose, sowie für allgemeine Einschränkungen in der körperlichen und kognitiven Funktionsfähigkeit [3–6].

Die Förderung gesunden Alterns wird damit zu einer zentralen Aufgabe von Public Health, Versorgungsforschung und kommunaler Planung. Entsprechend wurde „Gesund älter werden“ im Jahr 2012 als nationales Gesundheitsziel definiert und im Präventionsgesetz (§ 20 SGB V) verankert [7]. Zentrale Handlungsfelder sind die Stärkung der gesellschaftlichen Teilhabe, die Förderung körperlicher Aktivität und Mobilität sowie der Erhalt gesundheitlicher Ressourcen älterer Menschen. Diese Handlungsfelder verdeutlichen, dass gesundes Altern nicht allein durch medizinische Versorgung bestimmt wird, sondern wesentlich von alltagsbezogenen Verhaltensweisen und deren Umweltbedingungen abhängt [7].

**Abb. 1:**
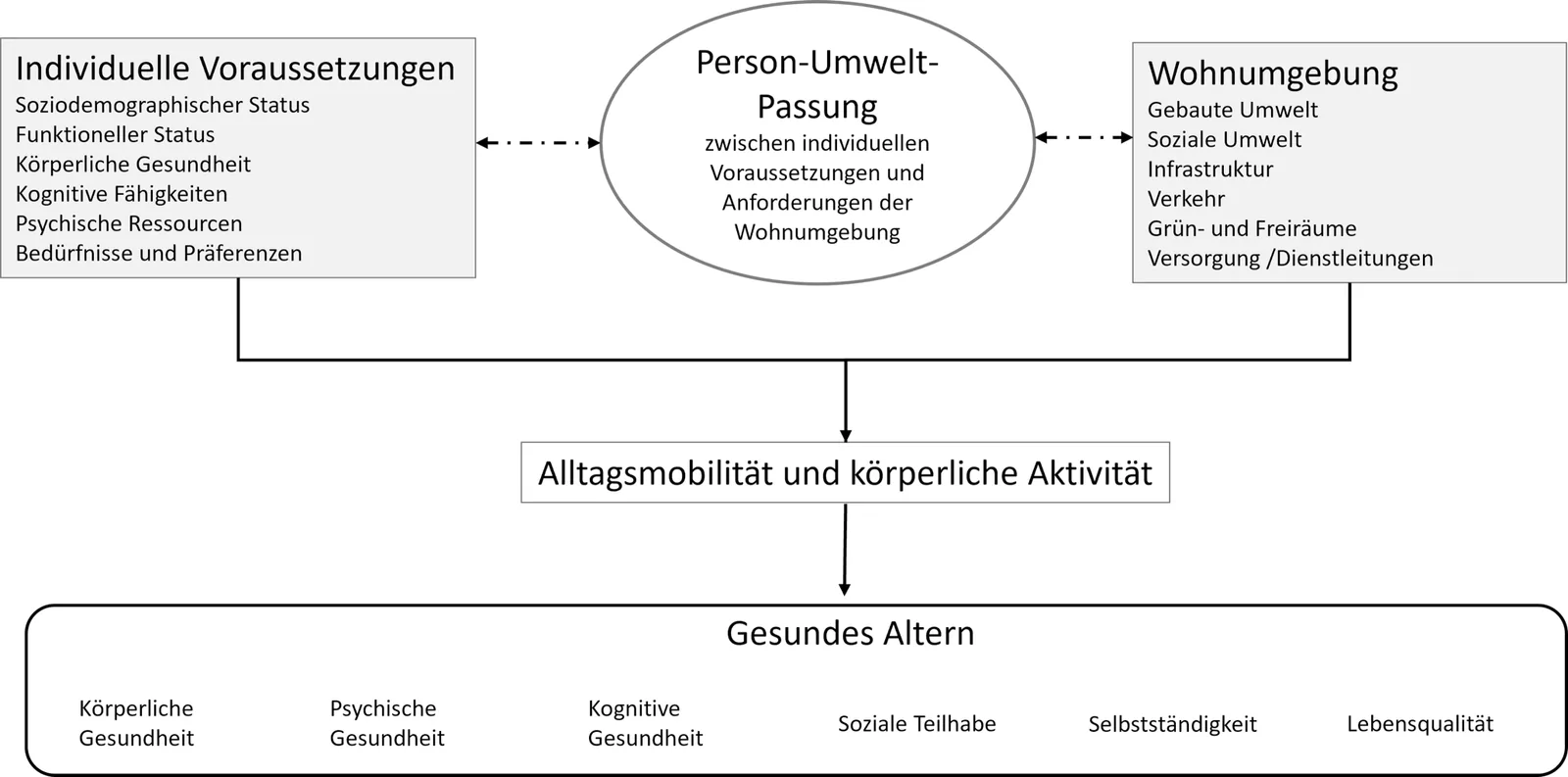
Konzeptionelle Grundlage für die Untersuchung von Wohnumgebung, Mobilität und gesundem Altern im höheren Lebensalter. Die Abbildung integriert Konzepte der Person-Umwelt-Passung nach Wahl et al. [8], den Zusammenhang zwischen Umwelt, Mobilität und Gesundheit nach Kerr et al. [9] sowie das multidimensionale Verständnis von Healthy Ageing der WHO

Zur konzeptionellen Einordnung dieser Zusammenhänge wird in Abb. 1 ein integriertes Modell dargestellt, das Konzepte der Umweltgerontologie [8] mit Public-Health-Ansätzen [9] hinsichtlich ihres Einflusses auf die Gesundheit im Alter [10] verbindet. Aus der Umweltgerontologie ist bekannt, dass die Wirkung der Wohnumgebung wesentlich von der Passung zwischen individuellen Voraussetzungen und Umweltanforderungen abhängt. Insbesondere funktioneller Status, gesundheitliche Ressourcen und individuelle Bedürfnisse beeinflussen, wie Umweltmerkmale wahrgenommen und genutzt werden. Die Person-Umwelt-Passung als Ergebnis der Interaktion zwischen individuellen Voraussetzungen und Umweltanforderungen bildet dabei die zentrale Voraussetzung für Mobilität und Teilhabe im höheren Lebensalter [8, 11]. Aufbauend darauf verdeutlicht das Modell nach Kerr et al. den vermittelnden Zusammenhang zwischen Wohnumgebung, Mobilität bzw. körperlicher Aktivität und gesundem Altern [9].

Die vorliegende konzeptionelle Grundlage integriert beide Perspektiven und dient als Ausgangspunkt für die Entwicklung eines Operationalisierungsframeworks für Walkability im höheren Lebensalter. Die Wirkung der Wohnumgebung auf Mobilität und Gesundheit erfolgt nicht unmittelbar, sondern wird sowohl durch die Passung zwischen individuellen Voraussetzungen und Umweltanforderungen als auch über das daraus resultierende Mobilitätsverhalten vermittelt.

Eigenschaften des Wohnumfelds können – abhängig von der Person-Umwelt-Passung – die Alltagsmobilität und körperliche Aktivität fördern oder behindern [9, 12–14]. Körperliche Aktivität nimmt dabei eine zentrale Rolle ein, da sie sowohl mit positiven Effekten auf die kardiovaskuläre, muskuloskelettale und psychische Gesundheit verbunden ist als auch präventiv gegen funktionelle Einschränkungen, Gebrechlichkeit und kognitive Beeinträchtigungen wirkt [15, 16]. Gleichzeitig beeinflusst sie positiv die Lebensqualität, unterstützt Selbstständigkeit, Autonomie und soziale Teilhabe im Alter [17–19].

Im internationalen Diskurs wird gesundes Altern nicht allein krankheitsbezogen verstanden, sondern als Erhalt funktionaler Fähigkeiten im Zusammenspiel mit der sozialen und physischen Umwelt. Damit rückt die Frage in den Vordergrund, unter welchen Bedingungen ältere Menschen im Alltag mobil bleiben, Wege selbstständig bewältigen und am gesellschaftlichen Leben teilhaben können. Die Weltgesundheitsorganisation (World Health Organization, WHO) hat diesen Zusammenhang im Rahmen der *Decade of Healthy Ageing* ausdrücklich hervorgehoben und betont, dass Gesundheit im Alter durch die Passung zwischen individuellen Ressourcen und Umweltbedingungen wesentlich mitbestimmt wird [20].

### Walkability als Merkmal des direkten Wohnumfelds

Gerade im höheren Lebensalter finden Mobilität, körperliche Aktivität, soziale Kontakte und Versorgung überwiegend im direkten Wohnumfeld statt. Die Gestaltung der physischen und sozialen Umwelt rückt damit als zentraler Einflussfaktor in den Fokus. Das direkte Wohnumfeld kann für körperliche Aktivität im Alter sowohl förderlich als auch hinderlich sein [12–14].

Dies wird mit dem Konzept der Gehfreundlichkeit, der Walkability, verdeutlicht: Walkability beschreibt den Grad, in dem die gebaute und soziale Umwelt das Gehen im Alltag unterstützt oder erschwert. Das Konzept umfasst verschiedene Dimensionen der Wohnumgebung, die die Erreichbarkeit von Zielen, Sicherheit und Attraktivität des Gehens beeinflussen. Walkability gilt als zentrales Konzept zur Beschreibung bewegungsförderlicher Wohnumgebungen [21, 22]. Walkability kann mit unterschiedlichen methodischen Ansätzen erfasst werden. Diese unterscheiden sich hinsichtlich der Art der Datenerhebung, der räumlichen Auflösung sowie der erfassten Umweltmerkmale. Einen systematischen Überblick über etablierte Erhebungsinstrumente zur Erfassung bewegungsförderlicher Umweltmerkmale im höheren Lebensalter geben Paulsen et al. [23]. Die wichtigsten methodischen Ansätze zur Erfassung von Walkability sind in Tab. 1 zusammengefasst. Objektive Verfahren erfassen Aspekte der gebauten Umwelt, wie die Vielfalt und räumliche Nähe unterschiedlicher Nutzungen wie Versorgung und Freizeit, ob Ziele gut zu Fuß erreichbar sind, welche Qualität die Bürgersteige haben oder ob es gute Möglichkeiten gibt, Straßen oder Schienen zu überqueren. Subjektive Verfahren erfassen Aspekte wie die wahrgenommene Sicherheit des Weges oder der Umgebung, die Aufenthaltsqualität eines Ortes und die Attraktivität der Umgebung [21, 24, 25]. Für ältere Menschen ist die Gehfreundlichkeit einer Wohnumgebung jedoch nicht ausschließlich eine Eigenschaft der gebauten oder sozialen Umwelt, sondern ergibt sich aus dem Zusammenspiel zwischen Umweltmerkmalen und den individuellen funktionellen Voraussetzungen der Person.

**Tab. 1:** Überblick über methodische Ansätze zur Erfassung von Walkability im höheren Lebensalter

Methodischer Ansatz	Beispiele für Verfahren	Erfassung	Stärken	Berücksichtigung der Person-Umwelt-Passung
GIS-/Index-basierte Verfahren	Walkability Index [21], Walk Score® (Redfin, Seattle, WA, USA), 15-Minute-City-Score	Objektiv, großräumig	Hohe Vergleichbarkeit, große Stichproben	Keine Berücksichtigung
Umwelt-Audits	SPACES [48], SWEAT [49], WRATS [50], SWAN [51]	Objektiv, kleinräumig	Detaillierte Erfassung von Gehwegen, Querungen, Barrieren und Sicherheitsaspekten	Teilweise indirekt (z. B. Barrierefreiheit, Gehwegbeschaffenheit)
Fragebogeninstrumente	NEWS [42], ALPHA [52], SWAN13 [51]	Subjektiv	Erfasst Wahrnehmungen, Sicherheitsempfinden und Attraktivität der Umgebung	Indirekt über individuelle Wahrnehmung
Qualitative Verfahren	Walk-Alongs, Walk-and-Talk-Interviews, Go-Along-Interviews, Community-Walking-Audits mit Bürgerbeteiligung	Subjektiv, kontextbezogen	Hohe Alltagssensitivität, Identifikation von Barrieren aus Nutzersicht	Direkte Erfassung individueller Einschränkungen und Umweltbarrieren
Partizipative Geoinformationsverfahren (PPGIS)	Public Participation GIS, Maptionnaire® (Mapita Oy, Helsinki, Finnland), Community Mapping	Subjektiv und objektiv	Verknüpfung räumlicher Informationen mit Nutzerperspektiven	Explizite Berücksichtigung der Nutzerperspektive, häufig auch funktioneller Einschränkungen

### Evidenz zu Walkability und körperlicher Aktivität im Alter

Studien mit älteren Menschen – je nach Studie überwiegend definiert als Personen ab 65 Jahren – zeigen, dass Merkmale der gebauten Umwelt körperliche Aktivität fördern. Fußgängerfreundliche Infrastruktur, wahrgenommene Sicherheit und die fußläufige Verfügbarkeit und Erreichbarkeit von Geschäften und Parks und deren Ästhetik wirken sich positiv auf körperliche Aktivität aus [26]. Trotzdem ist die Evidenz für die positiven Effekte von Walkability der Umgebung für ältere Menschen im Vergleich zu anderen Altersgruppen weiterhin nicht eindeutig. Kausale Zusammenhänge lassen sich aufgrund der überwiegend querschnittlichen Primärstudien nur eingeschränkt nachweisen [27]. Merkmale der physischen Umwelt wie Verkehrssicherheit, Kriminalität, Begrünung oder die Erreichbarkeit alltagsrelevanter Zielorte werden in Altersstudien häufiger untersucht. Da Studien mit jüngeren Bevölkerungsgruppen teilweise andere Schwerpunkte setzen, lässt sich nur eingeschränkt beurteilen, welche Zusammenhänge tatsächlich altersspezifisch sind [28]. Konsistent wird berichtet, dass für ältere Menschen die Erreichbarkeit von Orten, die den täglichen Bedarf decken, sowie Verkehrssicherheit und das subjektive Sicherheitsempfinden besonders wichtig sind [29, 30].

### Forschungslücken und Zielsetzung

Obwohl die Bedeutung der Person-Umwelt-Passung in der Umweltgerontologie seit Langem beschrieben wird, wird sie in der Operationalisierung bestehender Walkability-Ansätze bislang nur begrenzt berücksichtigt. Gerade im höheren Lebensalter hängt die Nutzbarkeit des Wohnumfelds nicht allein von dessen objektiver Struktur ab, sondern davon, wie der individuelle Gesundheitszustand mit den Aktivitäten des täglichen Lebens, dem Erreichen von Teilhabe, den Bedürfnissen und Eigenschaften der Person und den Gegebenheiten der Umwelt interagiert [31]. Wird die Person-Umwelt-Interaktion nicht angemessen berücksichtigt, besteht die Gefahr, dass Zusammenhänge zwischen Umwelt und körperlicher Aktivität verzerrt oder unvollständig erfasst werden. Funktionelle Einschränkungen werden hier im Sinne der Internationalen Klassifikation der Funktionsfähigkeit, Behinderung und Gesundheit (ICF) verstanden. Sie beschreiben Einschränkungen der Funktionsfähigkeit, die sich aus dem Zusammenspiel von Gesundheitszustand, Aktivitäten, Teilhabe sowie personenbezogenen und Umweltfaktoren ergeben und dadurch die selbstständige Nutzung des Wohnumfelds beeinflussen können [31].

Im Folgenden werden zunächst konzeptionelle Vorarbeiten und das entwickelte Operationalisierungsframework dargestellt, anschließend das WALK-Projekt als kommunale Anwendungsstudie beschrieben und abschließend Implikationen für Forschung und Praxis diskutiert.

## Entwicklung eines altersspezifischen Operationalisierungsframeworks für Walkability

Die etablierten Instrumente für Walkability sind nicht selektiv für bestimmte Altersgruppen konzipiert. Eigene Vorarbeiten aus der KORA-Age-Studie zeigten jedoch, dass ältere Menschen spezifische Anforderungen an die soziale und gebaute Umwelt stellen [32]. Barrieren wie schlechte Gehwege, fehlende öffentliche Toiletten oder große Distanzen zu Versorgungseinrichtungen schränken Mobilität und Teilhabe ein. Gleichzeitig bestehen Diskrepanzen zwischen objektiv vorhandener Infrastruktur und subjektiv wahrgenommenen Nutzungsmöglichkeiten.

In Fokusgruppeninterviews der Studie zeigte sich, dass soziale Teilhabe im Alltag eng mit wohnortnahen Aktivitäten wie körperlicher Bewegung, kulturellen Angeboten, ehrenamtlichem Engagement und familiären Aufgaben verknüpft ist. Zentrale Voraussetzung hierfür ist Mobilität, die sowohl individuell physisch als auch durch Umweltbedingungen geprägt wird. Neben gesundheitlichen Einschränkungen wurden insbesondere Sicherheitsaspekte, infrastrukturelle Defizite und eingeschränkte Erreichbarkeit von Versorgungs- und Dienstleistungsangeboten als Barrieren für Teilhabe identifiziert. Demgegenüber wirken soziale Beziehungen und nachbarschaftlicher Zusammenhalt als wichtige Ressourcen, die Teilhabe fördern.

Diese Ergebnisse verdeutlichen, dass Walkability im höheren Lebensalter nicht auf klassische stadtplanerische Dimensionen wie Dichte, Nutzungsmischung oder Konnektivität reduziert werden kann. Vielmehr gewinnen zusätzliche Merkmale der Umgebung an Bedeutung. Hierzu zählen Sitzgelegenheiten, die Sicherheit bei der Überquerung von Straßen, Barrierefreiheit, Oberflächenqualität und Aufenthaltsqualität. Entscheidend für die Nutzbarkeit der Wege des Wohnumfelds ist, ob sie auch bei funktionellen Einschränkungen oder bei Gebrechlichkeit zugänglich und bewältigbar bleiben. Das Konzept Walkability muss daher stärker auf zielgruppenspezifische Umgebungsmerkmale zugeschnitten werden [28].

Vor diesem Hintergrund wurde vom Autorenteam ein spezifisches Operationalisierungsframework für Walkability im höheren Lebensalter entwickelt. Es strukturiert und priorisiert Umweltindikatoren, um Walkability im höheren Lebensalter funktionssensitiv, also in Abhängigkeit vom funktionellen Status älterer Menschen, erfassen zu können. Es konzentriert sich auf solche Umweltmerkmale, die im Kontext von Walkability operationalisierbar sind. Hierzu gehören neben Merkmalen der gebauten Umwelt auch Walkability-relevante Aspekte der sozialen Umwelt, insbesondere Sicherheit, Attraktivität sowie die Erreichbarkeit von Begegnungsorten. In einer Delphi-Konsensstudie wurden in 3 Stufen aus bestehenden und etablierten Walkability-Instrumenten Indikatoren identifiziert, die für die Gehfreundlichkeit im Alter relevant sind. In einem weiteren Schritt wurde eine Expert:innenbefragung durchgeführt, um zu überprüfen, inwieweit einsetzende Funktionseinschränkungen bei den jeweiligen Indikatoren berücksichtigt werden sollten.

Das resultierende Indikatorenset umfasst 3 Dimensionen: (1) Wohnumfeld und Verkehrsinfrastruktur, (2) Erreichbarkeit von Zielen und Begegnungsorten sowie (3) Attraktivität und Sicherheit der Umgebung. Besonders hervorzuheben ist die systematische Berücksichtigung der Person-Umwelt-Passung. Aufbauend auf der konzeptionellen Grundlage werden Umweltmerkmale nicht isoliert bewertet, sondern hinsichtlich ihrer unterschiedlichen Relevanz in Abhängigkeit vom funktionellen Status älterer Menschen. Damit wird Walkability nicht nur als Eigenschaft des Raums, sondern als relationale Größe zwischen Person und Umwelt konzipiert.

Tab. 2 zeigt exemplarisch jene Umweltmerkmale des Operationalisierungsframeworks, deren Bedeutung für Mobilität und Teilhabe besonders stark von der individuellen Funktionsfähigkeit abhängt. Daher werden Umweltmerkmale der Dimension „Attraktivität und Sicherheit“ nicht gesondert dargestellt, da diese in der Delphi-Konsensstudie unabhängig vom funktionellen Status als relevant bewertet wurden.

**Tab. 2:** Umweltmerkmale mit erhöhter Relevanz für Walkability bei funktionellen Einschränkungen im Alter

Wohnumfeld und Verkehrsinfrastruktur	Straßenquerungen
	Ampelschaltungen
	Gut einsehbare Wege
	Geländer
	Sitzgelegenheiten
	Erreichbarkeit
	Gehsteige barrierefrei
	Ausreichend Schatten
Erreichbarkeit von Zielen und Begegnungsorte	Sitzmöglichkeiten
	Gut ausgestattete Haltestellen
	Öffentliche Toiletten
	Wohnortnahe Versorgung (inkl. medizinischer Versorgung)
	Verfügbarkeit von Bewegungsangeboten
	Soziale Begegnungsmöglichkeiten

Das entwickelte Indikatorenset bildet damit 2 wichtige Grundlagen für Anwendungsstudien für die wohnortnahe Bewegungsförderung im Alter ab: Es präzisiert erstens, welche Umweltmerkmale für ältere Menschen relevant sind, und schafft zweitens eine Grundlage für die kommunale Operationalisierung. Damit kann eine Lücke zwischen Walkability-Forschung, kommunaler Praxis und altersspezifischer Gesundheitsforschung geschlossen werden [33, 34].

## Anwendungsstudie: Bewegungsförderung und Walkability in München – das WALK-Projekt

Aufbauend auf den dargestellten konzeptionellen Vorarbeiten und dem entwickelten Framework für Walkability stellt sich die Frage, wie die entwickelten Indikatoren unter realen Bedingungen in einem urbanen Kontext angewendet und validiert werden können. Das Projekt WALK möchte diesen nächsten translationalen Schritt gehen: In einer konkreten urbanen Wohnumgebung soll untersucht werden, welche Indikatoren für ältere Menschen das Gehen im Alltag fördern oder behindern, wie diese Indikatoren von unterschiedlichen Gruppen älterer Menschen – beispielsweise in Abhängigkeit von Geschlecht, sozioökonomischem Status, Migrationsgeschichte oder funktionellem Status – erlebt werden und welche Schlussfolgerungen sich daraus für eine gesundheitsfördernde kommunale Gestaltung ableiten lassen. Die Studie wird 2026/2027 durchgeführt und ist gefördert durch den Nachhaltigkeitsfonds der Ludwig-Maximilians-Universität München (LMU). Die Durchführung der geplanten Anwendungsstudie erfolgt vorbehaltlich eines positiven Ethikvotums der zuständigen Ethikkommission.

Da sich das WALK-Projekt derzeit in der Vorbereitungsphase befindet, beschreibt der vorliegende Beitrag die konzeptionellen Grundlagen der geplanten Anwendungsstudie. Die konkrete methodische Ausgestaltung, insbesondere die funktionssensitive Operationalisierung der Walkability-Indikatoren und die Integration der verschiedenen Datenquellen, wird im weiteren Projektverlauf entwickelt und in späteren methodischen Publikationen dargestellt.

### München als Anwendungsraum

Als erste Testregion ist für die Umsetzung des Projekts München vorgesehen, da hier günstige Voraussetzungen für eine praxisnahe Umsetzung gegeben sind. Dazu zählen vorhandene kommunale Vorarbeiten im Bereich der gesundheitsfördernden Stadtentwicklung [35], bereits bestehende etablierte Kooperationen mit lokalen Entscheidungsträgern und damit ein erleichterter Zugang zu weiteren Projektpartnern [36] sowie einer Vielzahl an Stadtbezirken bzw. Stadtbezirksteilen mit unterschiedlicher soziodemografischer Struktur, Bebauung, Verkehrsanbindung und Grünflächenanteilen [37]. Zudem zeigt sich insbesondere für München der demografische Wandel klar ab: Es wird prognostiziert, dass im Jahr 2035 rund 24 % der Bevölkerung über 60 Jahre alt sein werden [38].

### Studiendesign und methodische Umsetzung

Die Umsetzung erfolgt in ausgewählten städtischen Testregionen, die gemeinsam mit kommunalen Partner:innen und stadtteilbezogenen Stakeholdern so gewählt sind, dass diese eine möglichst breite Variation an Bevölkerungsstruktur (Alter, Geschlecht, sozioökonomischer Status, Migrationsgeschichte, Funktionsfähigkeit) und Stadtteilstruktur (soziale Lage, Grünflächenanteil, Verkehrsaufkommen, Wohnbebauung, demografische Zusammensetzung) aufweisen.

Die Projektdurchführung wird durch das MRC-Framework (entwickelt vom britischen Medical Research Council) für komplexe Interventionen geleitet [39], während die begleitende Evaluation basierend auf dem RE-AIM-Framework (Reach, Effectiveness, Adoption, Implementation, and Maintenance) durchgeführt wird [40].

Die Studie verfolgt ein Mixed-Methods-Design und kombiniert qualitative Verfahren, wie Interviews mit älteren Bewohner:innen, alltagsnahe Begehungen im Sinne von Walk-&-Talk-Ansätzen, qualitative Befragungen lokaler Expert:innen mit quantitativen Verfahren wie Geoinformationssystem-(GIS-)gestützten Analysen zur Gehfreundlichkeit. Diese Kombination objektiver räumlicher Analysen mit der subjektiven Wahrnehmung älterer Menschen ermöglicht es, Diskrepanzen zwischen vorhandenen Umweltmerkmalen und deren tatsächlicher Nutzung bzw. Wahrnehmung systematisch zu identifizieren.

Dabei sind insbesondere die saisonalen klimatischen Bedingungen zu berücksichtigen, da Faktoren wie Schattenplätze oder vereiste oder schlecht geräumte Wege das alltägliche Gehverhalten maßgeblich beeinflussen [21, 24, 25, 41, 42]. Die Studiendauer von 18 Monaten gewährleistet, dass die Datenerhebung unter unterschiedlichen saisonalen und klimatischen Bedingungen durchgeführt wird und so das klimatische Spektrum abgedeckt werden kann.

Die im Rahmen der Vorarbeiten entwickelten Walkability-Indikatoren bilden die Grundlage für die Datenerhebung und -auswertung. Die Ergebnisse werden stadtteilübergreifend analysiert und räumlich aufbereitet, um Bereiche mit guter Gehfreundlichkeit sowie spezifische Verbesserungspotenziale zu identifizieren. Die Einschätzung der Gehfreundlichkeit basiert dabei auf der integrierten Betrachtung objektiver räumlicher Merkmale und der subjektiven Wahrnehmung älterer Menschen.

### Praktische Umsetzung

Über die Stadtteil-Partner:innen werden Bewohner:innen für die Studie rekrutiert, die zunächst zu ihrem alltäglichen Gehverhalten, sonstigen körperlichen Aktivitäten, Motivation und Gesundheitszustand befragt werden. Mit einer Auswahl an Personen werden Alltagsrouten abgegangen. Während dieses Spaziergangs kommentieren die Teilnehmenden, warum sie bestimmte Wege wählen oder meiden und wie sie ihre Wohnumgebung während der Bewegung wahrnehmen (Walk & Talk bzw. Go-Along- oder Walking-Interview; [43, 44]).

Übergreifend werden Expert:innen aus stadtteilbezogenen Einrichtungen durch leitfadengestützte Interviews nach ihrer Einschätzung zu Bewegungsförderung vor Ort gefragt. Potenzielle Partner:innen sind hier beispielsweise Alten- und Servicezentren, Nachbarschaftstreffs oder Gesundheitstreffs. Hier werden auch mögliche Kontakte zu eventuell schwer erreichbaren Bevölkerungsgruppen gesucht und versucht, die lokale Walkability unter Berücksichtigung der Bedürfnisse älterer Menschen mit möglichen einsetzenden oder vorhandenen Einschränkungen in der Funktionsfähigkeit zu bewerten.

Die Ergebnisse werden stadtteilübergreifend analysiert und GIS-basiert aufgearbeitet. Ziel ist es, Bereiche zu identifizieren, in denen das alltägliche Gehen gut ermöglicht wird, sowie solche, die Verbesserungspotenziale aufweisen. Auf Grundlage der Synthese der Ergebnisse werden konkrete Handlungsempfehlungen zur Verbesserung der Gehfreundlichkeit im Wohnumfeld für ältere Menschen entwickelt.

**Abb. 2:**
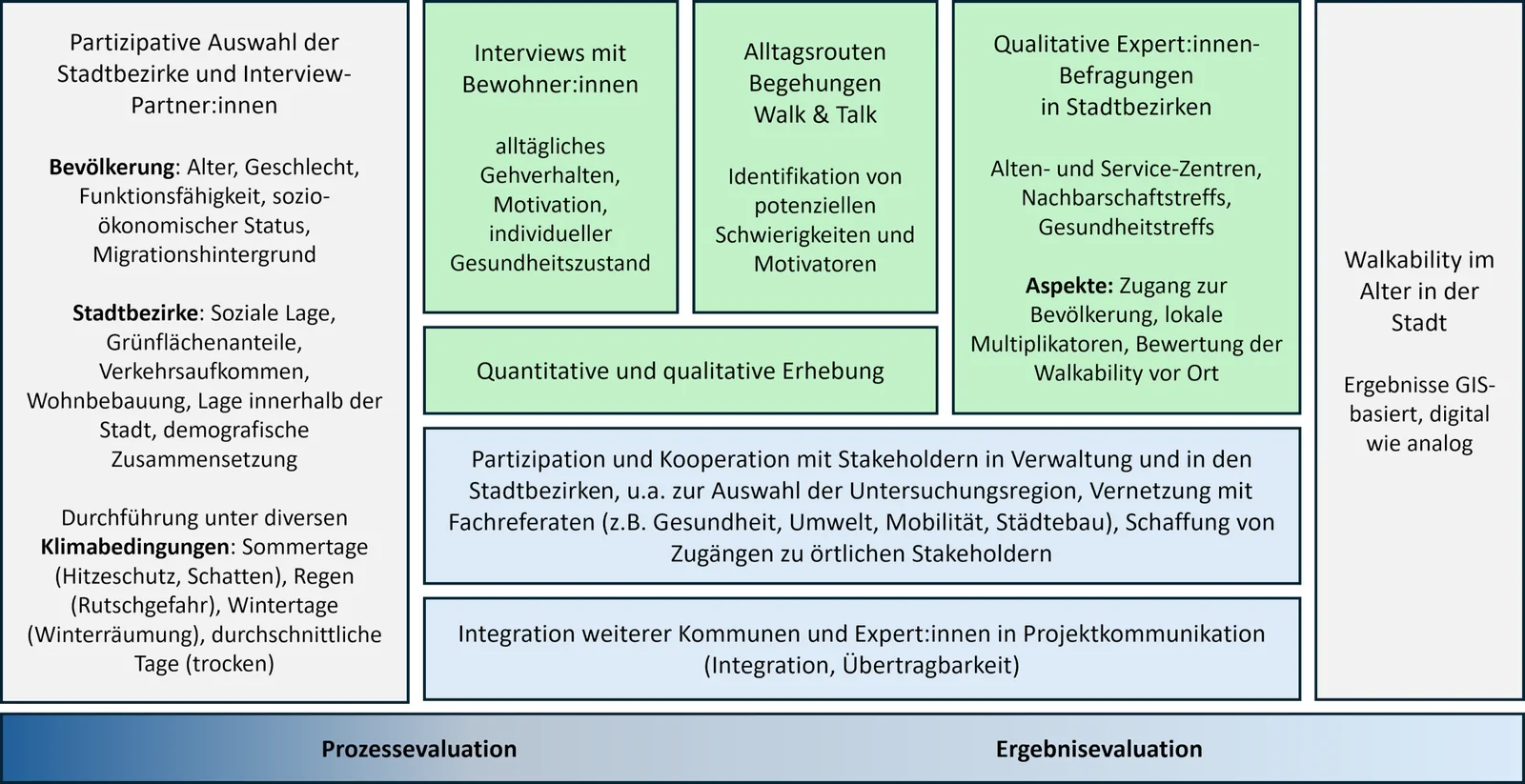
Studiendesign und methodisches Vorgehen im Projekt WALK

Abb. 2 veranschaulicht das Mixed-Methods-Design und die Verknüpfung von Datenerhebung, partizipativer Einbindung und räumlicher Analyse.

### Anschlussfähigkeit

Durch die konkrete Erprobung und Einbettung der Indikatorenliste in die Lebensrealität älterer Menschen und die Kooperation mit lokalen Expert:innen sollen förderliche Faktoren und Barrieren für wohnfeldnahe Bewegung und Teilhabe identifiziert und Empfehlungen für Interventionsmaßnahmen formuliert werden. Diese Empfehlungen können z. B. städtebaulich, aber auch sozialgesellschaftlich geprägt sein. Um die Übertragbarkeit und Anschlussfähigkeit sicherzustellen, werden von Beginn an weitere Städte und Gemeinden aktiv in den Projektprozess einbezogen. Die entwickelten Empfehlungen sollen nach Projektabschluss für andere Kommunen aufbereitet und zur Verfügung gestellt werden.

## Diskussion und weitere Schritte

Der Mehrwert des hier dargestellten Projekts liegt darin, dass Walkability nicht nur theoretisch oder kartografisch beschrieben, sondern an der Lebensrealität älterer Menschen gespiegelt werden soll. Dadurch kann geprüft werden, welche der im Operationalisierungsframework erarbeiteten Indikatoren sich in der Praxis tatsächlich als relevant erweisen, welche Barrieren bislang unzureichend erfasst sind und welche kommunalen Handlungsoptionen sich daraus ableiten lassen.

Die geplante kommunale Umsetzung wirft unmittelbar die Frage nach der Übertragbarkeit auf. Wir antizipieren, dass manche Ergebnisse stadtspezifisch sein werden, da Bebauungsstruktur, Topografie, Versorgungslage, Mobilitätskultur und kommunale Governance zwischen Städten variieren kann. Gleichzeitig spricht der bisherige Forschungsstand dafür, dass bestimmte Domänen von Walkability – etwa Erreichbarkeit, Sicherheit, Qualität der Wege und alltagsrelevante Zielorte – kontextübergreifend bedeutsam bleiben, auch wenn ihre konkrete Ausprägung und Gewichtung lokal unterschiedlich ausfallen. Diese Aspekte und die Einbeziehung der klimatischen Gegebenheiten können ebenso auf andere Kommunen übertragen werden. Darüber hinaus unterscheiden sich urbane und ländliche Räume hinsichtlich zentraler Walkability-Merkmale grundlegend. Insbesondere größere Distanzen zwischen alltagsrelevanten Zielorten sowie eine häufig fehlende oder unterbrochene Fußwegeinfrastruktur führen dazu, dass Walkability im ländlichen Raum anderen Rahmenbedingungen unterliegt. Das WALK-Projekt verfolgt daher bewusst das Ziel, ein Operationalisierungsframework im urbanen Kontext zu entwickeln und zu erproben. Die Übertragbarkeit auf ländliche Räume sollte Gegenstand zukünftiger Forschung sein.

Die Ergebnisse sollen daher systematisch für andere deutsche Kommunen aufbereitet, zur Verfügung gestellt und gemeinsam diskutiert werden. Eine systematische Übersichtsarbeit zu gebauter Umwelt und Gesundheit in Deutschland zeigte bereits, dass die nationale Forschungslage im internationalen Vergleich bislang begrenzt ist und kontextspezifische Analysen für deutsche Städte und Gemeinden notwendig sind [34].

Es gibt einige Erfahrungen aus anderen Städten, die auch in das WALK-Projekt einfließen sollen. Eine Studie hat kleinräumige Walkability-Unterschiede in den bevölkerungsreichsten deutschen Städten beschrieben und methodische Ansätze für eine an älteren Menschen orientierte Messung für 2 Städte entwickelt [45, 46]. Daneben liegt mit einer Mixed-Methods-Untersuchung in Regensburg eine neuere kommunal ausgerichtete Anwendung vor, die GIS-basierte Analysen mit Walk-Audits kombiniert und damit die Schnittstelle zwischen Datenanalyse und lokaler Begehung stärkt [47]. Diese Arbeiten zeigen, dass Walkability zunehmend als praktisch anschlussfähiges Konzept der gesundheitsorientierten Stadtplanung etabliert wird.

Es fehlt jedoch bislang eine breite etablierte, konsequent alterssensible Perspektive, die Funktionsfähigkeit systematisch einbezieht und zugleich auf kommunale Planung übertragbar ist. Das WALK-Projekt adressiert diese Lücke, indem es konzeptionelle Entwicklung, empirische Anwendung und partizipative Umsetzung miteinander verbindet. Mittelfristig kann auf dieser Grundlage ein alterssensibles Walkability-Konzept entstehen, das sowohl wissenschaftlich belastbar als auch für kommunale Praxis nutzbar ist. Für die kommunale Praxis ergibt sich daraus die Notwendigkeit, die identifizierten Faktoren in konkrete, priorisierbare Maßnahmen zu überführen und Walkability stärker als integralen Bestandteil gesundheitsorientierter Stadtentwicklung zu verankern. Hierzu zählen sowohl baulich-infrastrukturelle Interventionen als auch sozialräumliche Ansätze zur Förderung von Teilhabe. Langfristig könnte Walkability als indikatorbasierter Bestandteil kommunaler Planungs- und Monitoringprozesse etabliert werden.

## Fazit

Walkability im höheren Lebensalter sollte nicht allein als Eigenschaft des gebauten Raums verstanden werden, sondern als Ergebnis der Passung zwischen Umweltbedingungen und individueller Funktionsfähigkeit. Der vorliegende Beitrag zeigt, dass eine alterssensible und funktionsorientierte Weiterentwicklung des Walkability-Konzepts notwendig ist, um Mobilität, körperliche Aktivität und soziale Teilhabe älterer Menschen angemessen abzubilden. Mit dem vorgestellten Framework und der geplanten Anwendungsstudie wird ein Ansatz beschrieben, der konzeptionelle Entwicklung, kommunale Anwendbarkeit und partizipative Perspektiven verbindet. Damit wird eine Grundlage geschaffen, um Barrieren und Förderfaktoren des alltäglichen Gehens systematisch zu erfassen und in gesundheitsfördernde Maßnahmen der Stadtentwicklung zu überführen.

## Data Availability

Alle dieser Arbeit zugrunde liegenden Daten sind in diesem Artikel enthalten.
